# P-857. Impact of Cefoxitin Monotherapy Versus Traditional Antimicrobial Therapy on Time to Antibiotic Administration in Intra-amniotic Infections

**DOI:** 10.1093/ofid/ofaf695.1065

**Published:** 2026-01-11

**Authors:** Hannah Bischoff, Sarah Withers, Caroline Jozefczyk, Joseph Kohn, R Jake Crocker, Jasmine Lewis, Carolyn Ellison, Alex Ewing, Pamela Bailey, Pamela Bailey

**Affiliations:** Prisma Health Upstate - Greenville Memorial Hospital, Greenville, SC; Prisma Health, Greenville, South Carolina; Prisma Health - Upstate, Greenville, South Carolina; Prisma Health Midlands, Columbia, South Carolina; Prisma Health, Greenville, South Carolina; Prisma Health Greenville Memorial Hospital, Greenville, South Carolina; Prisma Health Richland, Columbia, South Carolina; Prisma Health-Upstate, Greenville, South Carolina; University of South Carolina School of Medicine, Columbia, South Carolina; University of South Carolina School of Medicine, Columbia, South Carolina

## Abstract

**Background:**

The American College of Obstetricians and Gynecologists (ACOG) recommends ampicillin and gentamicin as first-line therapy for intra-amniotic infections (IAI) with clindamycin for cesarean sections. Safety concerns with traditional antimicrobial therapies (TAT) include nephrotoxicity from gentamicin and *C. difficile* infections from clindamycin. Cefoxitin has a comparable spectrum of activity and is a recommended single-agent alternative. Studies have shown similar efficacy between cefoxitin and other treatments for IAI. This study addresses a research gap on timely treatment of IAI by comparing time to effective antibiotic administration between cefoxitin and TAT.

**Methods:**

This retrospective cohort study was conducted at a large, multi-site health system in South Carolina. The TAT group, treated with ampicillin and gentamicin, with or without clindamycin from 6/2022 to 5/2023, was compared to patients treated with cefoxitin from 6/2023 to 5/2024, following an update to institutional guidelines. All pregnant individuals aged 16 and older with diagnosed or presumed IAI were included.

**Results:**

A total of 300 patients were included, 150 each in TAT and cefoxitin group. Baseline characteristics were similar between TAT and cefoxitin patients. Vaginal delivery was the most common mode of delivery and rates of cesarean delivery were similar between groups. Most patients had a negative Group B *Streptococcus* screen. Receipt of antimicrobials within 60–90 minutes of order entry occurred in significantly more patients in the cefoxitin group compared to TAT group (69.3% vs. 4.7%, *p* < 0.001). Time to effective therapy was significantly shorter in the cefoxitin group (76.4 ± 93.3 vs. 183.7 ± 228.9 minutes, *p* < 0.001). There were no differences in mortality, 30-day infection-related readmission, or need for additional surgical/procedural intervention.

**Conclusion:**

Cefoxitin use for IAI significantly improves the time to effective antibiotic treatment, aligning with current guideline recommendations for prompt therapy. The absence of differences in secondary outcomes reinforces the clinical efficacy of cefoxitin, supporting its use as a first-line agent in the management of IAI.

**Disclosures:**

All Authors: No reported disclosures

